# Streptomyces anthophorae sp. nov. and Streptomyces nidicola sp. nov., novel actinobacteria isolated from a solitary bee

**DOI:** 10.1099/ijsem.0.007029

**Published:** 2026-01-29

**Authors:** Shawn M. Christensen, Martin Kaltenpoth, Heiko Vogel, Rachel L. Vannette

**Affiliations:** 1 Department of Entomology and Nematology, University of California-Davis, Davis, CA, USA; 2 Department of Insect Symbiosis, Max Planck Institute for Chemical Ecology, Jena, Germany

**Keywords:** actinobacteria, insect, *Streptomyces*, symbiosis

## Abstract

Six novel actinobacterial strains (BH034^T^, BH055, BH097, BH104, BH105 and BH106^T^) were isolated from developing brood in nests of the solitary bee species *Anthophora bomboides* from Bodega Bay, California, USA. Phylogenetic analysis based on a five-gene multilocus sequence alignment and whole-genome data positions all six strains within the genus *Streptomyces*, with close relation to *Streptomyces endophyticus* YIM 65594^T^ and *Streptomyces fractus* MV32^T^. Through genetic and chemotaxonomic analysis, five of the isolates (BH034^T^, BH055, BH097, BH104 and BH105) were found to be a clade representing one species [>96% average nucleotide identity (ANI)], whereas BH106^T^ was a distinct species (<93% ANI with each of the other isolate genomes). Within this species (BH034^T^-BH105), the genomes comprised on average 9.6 Mb (±0.4 Mb), encoded 8,640 (±349) predicted genes and had a G+C content of 70.9 (±0.07) mol%. The type strain, BH034^T^, contained iso-C_16 : 0_, anteiso-C_15 : 0_ and iso-C_15 : 0_ as major fatty acids and contained ll-diaminopimelic acid in the cell wall. The remaining strain, BH106^T^, represents a distinct species; its genome comprised 9.4 Mb, encoded 8,426 predicted genes and had a G+C content of 70.7 mol%; the major fatty acids were anteiso-C_15 : 0_, anteiso-C_17 : 0_, iso-C_17 : 0_ and iso-C_15 : 0_, and the cell wall also contained ll-diaminopimelic acid. Functional genomic analysis revealed multiple secondary metabolite gene clusters in the bee-associated *Streptomyces* strains, several of which were found to be absent in closely related *Streptomyces* species. Based on genotypic, phenotypic and chemotaxonomic analyses, strains BH034-BH105 and BH106 represent two novel species within the genus *Streptomyces*, for which the names *Streptomyces anthophorae* sp. nov. (type strain BH034^T^=NRRL B-65741^T^=DSM 119658^T^) and *Streptomyces nidicola* sp. nov. (type strain BH106^T^=NRRL B-65742^T^=DSM 119659^T^) are proposed.

## Introduction

The genus *Streptomyces* (*Actinomycetota*) encompasses a diverse, ecologically complex and economically important group of bacteria [1,3]. *Streptomyces* are best recognized for their production of a multitude of bioactive natural products, including compounds with antibiotic, antifungal, anticancer, antiparasitic and immunosuppressive properties [4]. *Streptomyces*-derived antibiotics were among the first to be discovered (e.g. streptomycin), and many are still widely used to treat a variety of infections in humans as well as in crops and domesticated animals [3,5].

This plethora of secondary metabolites correlates with the genetic and ecological versatility and diversity within the genus *Streptomyces*. Members typically have large genomes, 6–12 Mb, as compared to ~5 Mb for the average bacteria [1,6]. These large genomes almost always include a number of biosynthetic gene clusters (BGCs), which are sets of genes that make the enzymatic ‘machinery’ to produce secondary metabolites [5]. These metabolites can have beneficial functions, such as eliminating competing microbes, scavenging iron, manipulating a host, attracting organisms for spore dispersal or signalling to other members of the same or other species [7]. The BGCs and the secondary metabolites they produce can vary considerably between species, and for many of these metabolites, the ecological roles are yet unknown [1].

*Streptomyces* typically grow as multicellular vegetative hyphae (filamentous) with highly coordinated metabolism, phenology and responses to the environment. They are generally nonmotile and produce spores for dispersal to new habitats. While they are highly prevalent and abundant in soil, *Streptomyces* can also be symbionts of multicellular eukaryotes, especially plants and invertebrates [8,10]. Specifically, certain insects, including beewolf wasps, bark beetles and ants, are associated with *Streptomyces* bacteria that produce bioactive secondary metabolites and may aid in the defence of the insects’ progeny or nutritional resources against pathogens [11,13].

In previous work, we isolated four *Streptomyces* strains from samples of the solitary bee *Anthophora bomboides* during development in the brood cell [14]. Based on amplicon sequencing, the genus *Streptomyces* was determined to be a core taxon within the developing brood cell of *A. bomboides,* and its abundance increased dramatically during the host overwintering period, when bee immunity is suppressed and fungal infections are most prevalent. Isolated strains were found to inhibit fungal growth to various degrees and were abundant in bee brood cell habitats, prompting further investigation. The strains isolated and sequenced in this study match two of the three most common bee-associated *Streptomyces* amplicon sequencing variants (ASVs) that were found in over half of the brood cells [14]. Additionally, the strains sequenced here were isolated from different brood cells, in three different developmental stages and seasons, and during three different years (Table 1), yet five of the six represent strains of the same species. Although it is possible that the bees are acquiring these ASVs from environmental sources that we did not previously sample, the combination of consistent *Streptomyces* presence*,* low overlap of ASVs between brood cells and environment, high prevalence of ASVs matching the sequence of the isolates and consistent re-isolation of *Streptomyces anthophorae* sp. nov. from the brood cells over time suggests a symbiotic association of these bacteria with *A. bomboides*.

**Table 1.** Strain sample collection metadata The table shows each strain, the proposed species characterization, the source from which the strain was isolated (all various developmental stages of *A. bomboides*) and the date on which the sample was collected. All strains were isolated from different brood cells. Superscript T indicates type strains of each proposed species.

Strain	Proposed species	Source	Collection date
BH034^T^	*S. anthophorae* sp. nov.	Early pollen provision	4 June 2021
BH055	*S. anthophorae* sp. nov.	Egg stage pollen provision	9 June 2021
BH097	*S. anthophorae* sp. nov.	Overwintering prepupa	25 October 2022
BH104	*S. anthophorae* sp. nov.	Overwintering prepupa	25 October 2022
BH105	*S. anthophorae* sp. nov.	Pupa	12 May 2023
BH106^T^	*Streptomyces nidicola* sp. nov.	Pupa	12 May 2023

Here, we report on the genomes of the four previously cultivated and two additional *Streptomyces* strains, each of which was isolated from distinct brood cells containing larval provisions, prepupae or pupae of *A. bomboides*. Based on our findings, we describe two new species, *S. anthophorae* sp. nov. and *Streptomyces nidicola* sp. nov., and provide their genomic sequences in comparison to their closest relatives.

## Methods

### Isolation and cultivation

Samples of the solitary bee *A. bomboides* were collected from Bodega Head Marine Reserve (SCSP permit issued 24 February 2020, 38.304273081815936, –123.06436189200674). Six isolates were obtained from various stages of *A. bomboides*’ life cycle (Table 1). These brood cells were collected and then *Streptomyces* were isolated from samples via repeated streak plating on tryptic soy agar (TSA) with cycloheximide (grown at 28 °C for 5–7 days). These isolates underwent preliminary identification via 16S rRNA gene Sanger sequencing as described in [14]. Once single-colony isolates were obtained, these were cryopreserved as glycerol stocks at −70 °C.

### Phenotypic characterization

#### Morphology

To determine morphological characteristics, each strain was plated from freezer stock onto multiple media types [TSA, maltose yeast media (MYM) and oatmeal agar (OA); see Table S1, available in the online Supplementary Material] and inoculated into liquid media [supplemented Grace’s media (SGM), Gibco] and then observed every other day for 7 days, noting colony colour, changes in media colour (diffusible pigments), colony shape, colour and surface texture. Colours were described according to the ISCC-NBS colour standard [15]. A dissection microscope was used to examine whether the strain produced spores on each media type. From liquid media, 10 µl was spotted onto a glass slide every other day for 7 days to determine morphological changes in growth, especially fragmentation of the filaments, as this trait was observed to differ between close relatives *Streptomyces fractus* and *Streptomyces endophyticus* [16,17].

#### Salt tolerance

Salt tolerance has been observed in related species *S. fractus* and was thus tested for all available strains [17]. To test salt tolerance, SGM was modified with various concentrations of sodium chloride (NaCl) at 0%, 5%, 10% and 15% w/v. Isolates were grown on TSA plates, and one colony (1 µl loop) was added to 5 ml of each media. Growth was determined after 5 days by increased OD after resuspension via agitation, as compared to the control (media without inoculation).

#### Temperature range

Each isolate was plated on TSA plates from freezer glycerol stocks; these were kept at 5 °C in a refrigerator and 25, 28, 37, 40 and 42 °C in incubators. Plates were monitored for growth every other day. Only type strains were tested at 5 and 42 °C.

#### Geosmin production

Geosmin production is a common trait for *Streptomyces* species and is what gives soil its ‘earthy’ smell [18]. After initial observations that BH106^T^ was less distinctly ‘earthy’ smelling than the other new isolates, geosmin production was inferred for each strain. The isolates were plated on TSA and allowed to grow for 5 days wrapped in parafilm; then, each plate was opened and the presence of geosmin was inferred by scent. This was repeated twice. Capacity for geosmin production was confirmed for all isolates via genome analysis (Table S3).

#### Substrate utilization

To determine the range of substrates each species could use, we purchased Biolog GEN III Microplates to determine the growth of the isolates on 71 different substrates. The type strain of each proposed species (BH034^T^ and BH106^T^) and the related *S. fractus* MV32^T^ and *Streptomyces kunmingensis* DSM 41681^T^ were grown on TSA plates for 5 days at 28 °C, at which time colonies were picked, broken up with a sterile pipette tip in a sterile tube, vortexed in 500 µl of sterile 1% NaCl solution and left for 60 min for larger pieces to settle out of solution. The upper suspension was then added to an additional 1% NaCl solution to an initial OD600 between 0.005 and 0.007. These were kept at 28 °C for 5 days, at which time each well was assessed for growth using an inverted microscope. This procedure was completed twice for each strain.

### Chemotaxonomy

The type strain for each proposed species (BH034^T^ and BH106^T^) was grown on TSA for 3 days at 28 °C. Biomass was scraped gently from the surface of the media, washed in sterile molecular grade water and resuspended in isopropanol for submission. Analyses for cellular fatty acids and the isomer of 2,6-diaminopimelic acid were carried out by DSMZ Services, Leibniz-Institut DSMZ-Deutsche Sammlung von Mikroorganismen und Zellkulturen GmbH, Braunschweig, Germany, following the protocols of [19,21].

### Genome sequencing and analysis

DNA was extracted from isolated strains using the Qiagen PowerSoil Pro kit; then, DNA concentration was assessed via NanoDrop. Sanger sequencing of the 16S rRNA gene using universal bacterial primers (27F/1492R) confirmed that the strains belong to *Streptomyces* using NCBI blast (16S ribosomal RNA sequence database) [22,23]. The genomes were sequenced using Pacific Bioscience (PacBio, Sequel 2 using 8M chips) long-read sequencing at the Integrated Microbiome Resource of Dalhousie University [24] and assembled with Flye (v2.9.3) [25]. The quality and completeness were calculated with CheckM (v1.2.2), which determined completeness of >99% for five genomes, and over 98.5% for one genome [26]. All bee isolate genomes had very low contamination, max 1.29% as determined by CheckM. Coverage ranged from 29× (BH097) to 419× (BH105) (mean=223× and median=205×). The genome sizes ranged from 8.75 Mb (BH104) to 9.67 Mb (BH097) with G+C content ranging from 70.7 to 71.0%. For details, see Table 2.

**Table 2.** Genome sequencing assembly results and quality The table shows each strain, the proposed species characterization, genome size in Mb, the G+C content (mol%), completeness (%) and contamination (%) as determined by CheckM, number of predicted genes, coding density as a proportion, the average fold coverage of the genome, number of contigs, N50 of assembly scaffolds in Mb, number of tRNAs, number of copies of rRNA, the Whole Genome Shotgun (WGS) accession and BioSample of the genomes on NCBI and, for each type strain, the collection ID for USDA ARS and DSMZ culture collections.

Proposed species	*S. anthophorae* sp. nov.					*S. nidicola* sp. nov.
Strain	BH034^T^	BH055	BH097	BH104	BH105	BH106^T^
**Genome size (Mb**)	9.62	9.66	9.67	8.76	9.67	9.46
**G+C content (mol%**)	71	70.9	71	70.9	70.9	70.7
**Completeness (%**)	99.89	99.46	99.46	99.89	99.46	98.61
**Contamination (%**)	1.29	1.29	0.43	1.29	1.29	0.43
**Predicted genes**	8,716	8,797	8,774	8,026	8,891	8,426
**Coding density**	0.894	0.892	0.893	0.893	0.894	0.895
**Fold coverage avg**.	80	196	29	214	419	400
**Contigs**	3	3	6	6	4	2
**N50 scaffolds (Mb**)	9.38	9.42	9.4	6.14	9.56	9.43
**tRNAs**	65	65	67	68	65	65
**rRNA copies**	7	7	7	7	7	7
**WGS accession**	JBLWQQ000000000	JBLWIQ010000000	JBNBPK000000000	JBMHXW000000000	JBNBPJ000000000	JBNBPI000000000
**BioSample genome**	SAMN47132431	SAMN46926878	SAMN47438835	SAMN47438836	SAMN47438837	SAMN47438838
**16S rRNA accession**	PP576370	PP576373	PP576371	PP576372	PV476755	PV476756
**Collection ID USDA ARS**	NRRL B-65741					NRRL B-65742
**Collection ID DSMZ**	DSM 119658					DSM 119659

Similarity to known strains was determined in several ways. The full 16S rRNA gene was extracted from the genomes (as annotated by Prokka), and NCBI blast was then used to determine closest potential relatives [22,27]. This determined that *S. fractus* isolated from the termite gut (*Amitermes hastatus*) was a close relative, but the species did not yet have a genome sequence [17]. We therefore ordered the strain (MV32^T^) from the USDA ARS Culture Collection and sequenced it using Oxford Nanopore Technology at the Max Planck Institute for Chemical Ecology. Sequencing was done on a MinION Mk1B device using a R10.4.1 flow cell, producing 11 Gb of data. Super high-accuracy basecalling of the raw reads was performed with Guppy v6.0.1 (Nanopore Technologies), and the *S. fractus* MV32^T^ genome was assembled with Flye (v2.9.2) [25], setting the minimum overlap as 10 kb and using the ‘--meta’ option. Subsequently, four rounds of polishing with Racon (v1.3.3) [28] were performed starting from the Flye assembly. After each polishing round, reads were re-aligned to the resulting assembly with minimap2 (v2.17) [29]. A final round of polishing was performed using Medaka (v1.2.0) [30]. The final genome assembly had a mean coverage of 1,100. Quality and completeness of the *S. fractus* MV32^T^ genome were calculated as above; the genome is 99.89% complete, with 1.65% contamination (NCBI accession number: JBMYHI000000000).

Digital DNA–DNA hybridization (dDDH) was used to estimate the genetic relatedness between bacterial genomes based on the digital analysis of genomic sequences. It serves as a digital approximation of the traditional DNA–DNA hybridization method, which is used to determine genomic similarity and to classify bacterial species, with d0 <70% indicating species separation [31]. The DSMZ type strain genome server was used for dDDH with their continuously updated library of known genomes to determine genome overlap/relatedness among the strains and other previously sequenced *Streptomyces* [32,33]. It uses dDDH to compare the genomes for relatedness and generated a whole-genome phylogeny using D5 distance formula and ‘GreedyWithTrimming’ distance algorithm, while the 16S rRNA gene phylogeny used the ‘CharacterCoverage’ distance algorithm [32]. Determination of closest type strain genomes was done in two complementary ways: First, all uploaded genomes were compared against all type strain genomes available in the TYGS database via the MASH algorithm, a fast approximation of intergenomic relatedness, and the ten type strains with the smallest MASH distances chosen per user genome [34]. Second, an additional set of ten closely related type strains was determined via the 16S rRNA gene sequences. These were extracted from the user genomes using RNAmmer, and each sequence was subsequently BLASTed against the 16S rRNA gene sequence of each of the 21,293 type strains available in the TYGS database on 10 June 2024 [35]. This was used as a proxy to find the best 50 matching type strains (according to the bit score) for each uploaded genome and to subsequently calculate precise distances using the Genome blast Distance Phylogeny (GBDP) approach under the algorithm ‘coverage’ and distance formula d5. These distances were finally used to determine the 10 closest type strain genomes for each of the uploaded genomes.

#### Pairwise comparison of genome sequences

For the phylogenomic inference, all pairwise comparisons among the set of genomes were conducted using GBDP and accurate intergenomic distances were inferred under the algorithm ‘trimming’ and distance formula d5 [36]. One hundred distance replicates were calculated for each comparison. dDDH values and confidence intervals were calculated using the recommended settings of the GGDC 4.0.

#### Phylogenetic inference

**Fig. 1.**
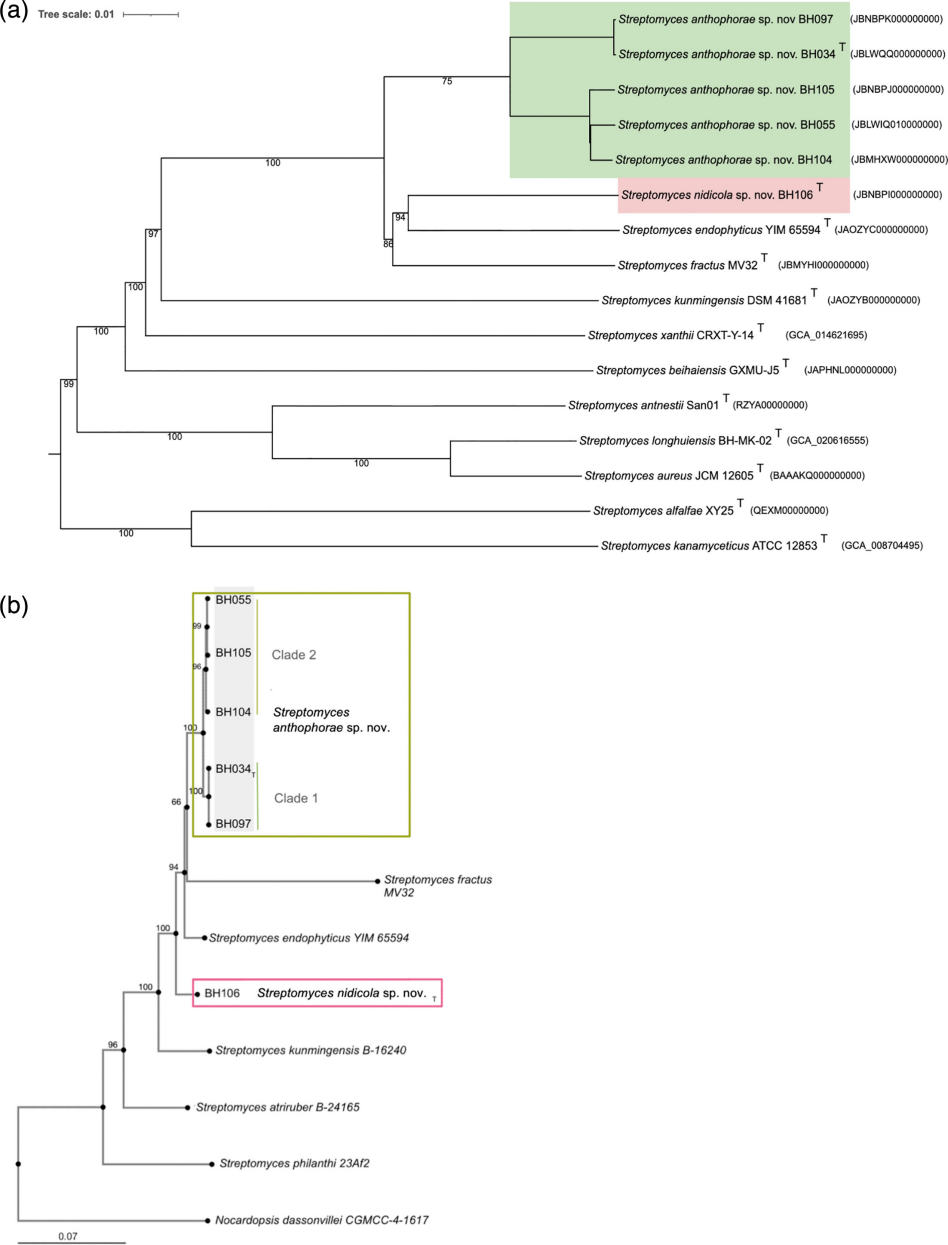
Phylogenetic position of the bee-associated species *S. anthophorae* sp. nov. and *S. nidicola* sp. nov. within the genus *Streptomyces*. (a) Genome tree created on DSMZ server, inferred with FastME 2.1.6.1 from GBDP distances calculated from genome sequences. The branch lengths are scaled in terms of GBDP distance formula d5, scale at top left. The numbers at nodes are GBDP pseudo-bootstrap support values >70% from 100 replications. New species are represented by coloured boxes and show distinction for the two new proposed species. Tree formatted in iTOL [67]. (b) Five-gene multilocus sequence alignment was performed using genes commonly used for phylogeny in existing *Streptomyces* literature (*atpD*, *gyrB*, *recA*, *rpoB* and *trpB*), alignment performed on MAFFT with MAFFT-L-INS-i. Conserved sites (2,278) were then used to construct a neighbour-joining tree using the Jukes–Cantor model. The numbers at nodes indicate bootstrap support values >60% from 1,000 replications. Green rectangle (top) outlines the clade of five isolates representing *S. anthophorae* sp. nov. with the two clusters labelled as clade 1 and clade 2. The red rectangle (centre) indicates the isolate representing *S. nidicola* sp. nov. Branch length indicates base changes per 100 bases, scale bar in bottom left.

The resulting intergenomic distances were used to infer a balanced minimum evolution tree with branch support via FastME 2.1.6.1 including SPR postprocessing [37]. Branch support was inferred from 100 pseudo-bootstrap replicates each. The trees were rooted at the midpoint and visualized with PhyD3 [38]. The type-based species clustering used a 70% dDDH radius around each of the 54 type strains [32]. The resulting groups are shown in Fig. 1a.

#### Average nucleotide identity

Average nucleotide identity (ANI) is a widely used metric for estimating the genetic similarity between bacterial genomes. It provides a quantitative measure of the ANI shared between the aligned regions of two genomic sequences and is commonly used in microbial taxonomy and genomics to distinguish species [39]. We utilized fastANI (v1.34) to determine ANI among the isolates and with other known genomes, using a cutoff of 95% to determine species-level relatedness [39].

#### Annotation

The genomes of the *Streptomyces* isolates were annotated with Prokka v1.14.6 [27]. Prokka was executed on the UC Davis FARM high-performance computing cluster, with default settings, the kingdom argument was set to ‘Bacteria’ and the genus argument set to ‘Streptomyces’, to optimize processing time. Prokka employs Prodigal for predicting protein-coding sequences, RNAmmer and Aragorn for identifying rRNA and tRNA genes and HMMER for small non-coding RNA detection [35,40,42]. Functional annotations utilize homology searches against UniProt and RefSeq databases [43,45]. Additionally, functional domains are annotated by searching against the Pfam database using HMMER [46]. From Prokka output, the Ghost Koala server was used to assign KO numbers to the amino acid sequences, and these were fed to KEGG Mapper to group genes into modules and pathways [47,48]. This annotation was also used for extracting multilocus sequence alignment (MLSA) gene sequences.

The genomes were analysed for BGCs using antiSMASH web server (antibiotics and Secondary Metabolite Analysis SHell, v7.0), a specialized software tool designed for the identification and annotation of secondary metabolite BGCs [49]. The analysis was conducted using the default settings of antiSMASH, which was configured to include all cluster types. AntiSMASH employs a combination of rule-based heuristics and profile hidden Markov models to predict and annotate BGCs, as well as algorithms to identify putative novel gene clusters by comparing them with known clusters in the Minimum Information about a Biosynthetic Gene cluster (MIBiG) repository [50].

The output from antiSMASH included a comprehensive list of predicted BGCs with annotations, including cluster types, core biosynthetic genes and additional tailoring and transport genes. The results were manually curated and visualized using the antiSMASH interactive HTML output, followed by export and integration with results from related genomes.

#### Multilocus sequence alignment

A five-gene MLSA was performed using genes commonly used for phylogeny in existing *Streptomyces* literature (*atpD, gyrB, recA, rpoB* and *trpB*) [51,52]. The five genes were extracted from the Prokka annotation files of each isolate and reference genome and concatenated. Multiple sequence alignments were performed using Multiple Alignment using Fast Fourier Transform (MAFFT, v7.0) online server to align the concatenated sequences [53]. The alignment parameters were configured to include the default settings unless otherwise specified. The ‘Auto’ strategy was initially used to allow MAFFT to select the appropriate algorithm based on sequence length and similarity and picked MAFFT-l-INS-i (most accurate). Following alignment, a phylogenetic tree was constructed using conserved sites (2,278) with the neighbour-joining (NJ) method within MAFFT. MAFFT computed the distance matrix using the Jukes–Cantor (JC69) model and resampling level of 1,000 for bootstrap values.

## Results

Genome sequencing and analysis showed that the six new bee-isolated strains represent two novel *Streptomyces* species. This is based on whole-genome comparison methods (dDDH, ANI) as well as MLSA and subsequent phylogenetic analyses, combined with comparative morphology and physiology [54].

### Genome comparisons

#### Phylogeny

The separation of the new bee isolates into two novel species is supported by the whole-genome GBDP tree and the five-gene MLSA NJ tree (*atpD, gyrB, recA, rpoB* and *trpB*), which both group BH034-BH105 into one clade, with BH106^T^, *S. fractus* MV32^T^ and *S. endophyticus* YIM 65594^T^ as closest neighbours (Fig. 1). The two trees are discordant in specific arrangement of BH106^T^, *S. fractus* MV32^T^ and *S. endophyticus* YIM 65594^T^; the genome tree has them together in a clade sister to BH034-BH105, with BH106^T^ more closely related to *S. endophyticus* YIM 65594^T^ (Fig. 1a)*,* whereas the MLSA separates them, with *S. fractus* MV32^T^ being sister to BH034 -BH104, *S. endophyticus* YIM 65594^T^ and BH106^T^ branching off earlier (Fig. 1b). In both cases, however, the five isolates representing *S. anthophorae* sp. nov. (BH034-BH105) form a monophyletic clade, with two subclades (BH034^T^/BH097 and BH055/BH104/BH105).

#### Whole-genome comparisons

dDDH was used to estimate the genetic relatedness between bacterial genomes, with d0 <70% indicating species separation [31]. ANI was also used to distinguish species, with a cutoff of 95% for species separation [55].

dDDH showed that the new strains have d0 <70% to any named species with a genome sequence available in the DSMZ database (Table S2) [33]. The closest dDDH matches were *S. endophyticus* YIM 65594^T^ which had a d0 of 67.7% with BH106^T^ and *S. fractus* MV32^T^ which had a d0 of 55.9% with BH034^T^ (Table S2) [16,17]. Additionally, the ANIs between these strains and other named species were less than the accepted species boundary of 95% ANI, as the closest named species genome was *S. endophyticus* YIM 65594^T^ with an ANI of 93.4% with BH106^T^ and *S. fractus* MV32^T^ with an ANI of 92.8%, also with BH106^T^, while all of the other new strains were below 93% ANI with any species with previously sequenced genomes.

**Fig. 2.**
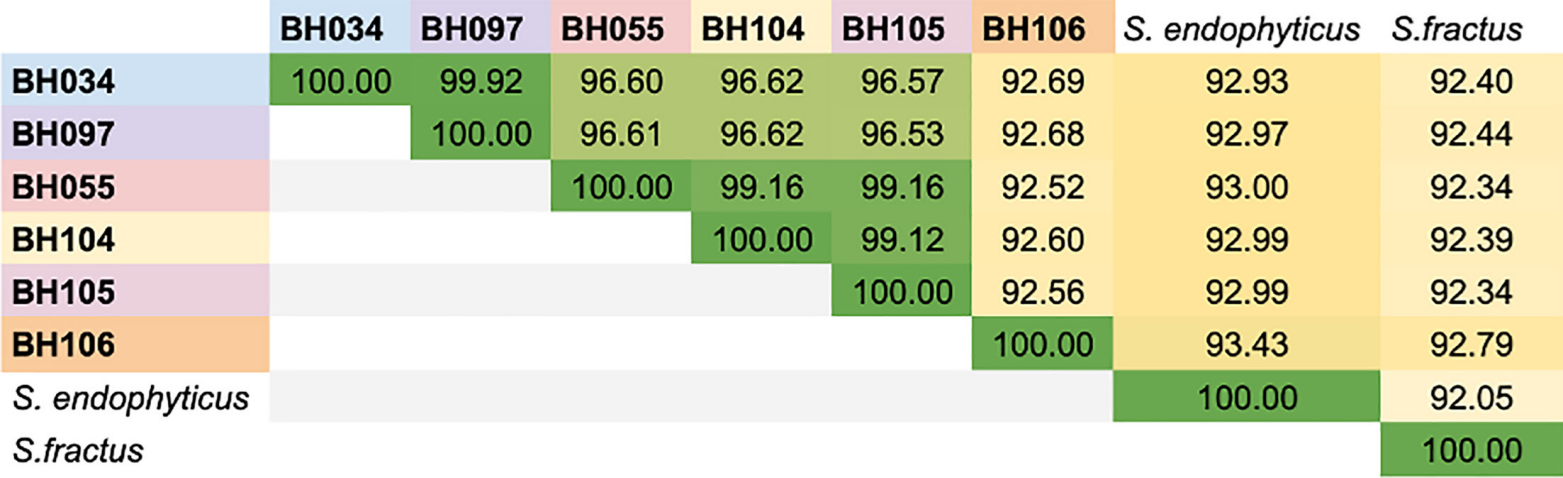
ANI between strains of *S. anthophorae* sp. nov., *S. nidicola* sp. nov. and closest relatives. Whole-genome ANI comparisons between strains of *S. anthophorae* and *S. nidicola* and the two most closely related previously described species (*S. endophyticus* YIM 65594^T^ and *S. fractus* MV32^T^). ANI was calculated using fastANI (v1.34). Numbers represent the ANI value of the genome in the row as compared to the column. The colour of boxes with ANI values is on a gradient from yellow (lower) to dark green (higher) ANI. The cutoff for species level is 95% ANI.

When comparing dDDH and ANI among the new strains, we find that two distinct species are present, with BH034^T^, BH055, BH097, BH104 and BH105 clustering together in one clade (>96% ANI), whereas BH106^T^ has <93% ANI with each of the other five genomes (Fig. 2). dDDH was also supportive of these two species, as within-clade d0 >70% (aside from BH104 which had d0 of 69% with BH034^T^ and BH097), but comparing BH106^T^ to the other new strains showed d0 between 60 and 66% (Table S2). At an even finer scale, the first species can be subdivided into two subspecies clades, with BH034^T^ and BH097 being nearly identical (ANI 99.9%, d0 99.8) and BH055, BH104 and BH105 having over 99% ANI and d0 of 80–90.9 with one another.

#### BGC comparisons

**Fig. 3.**
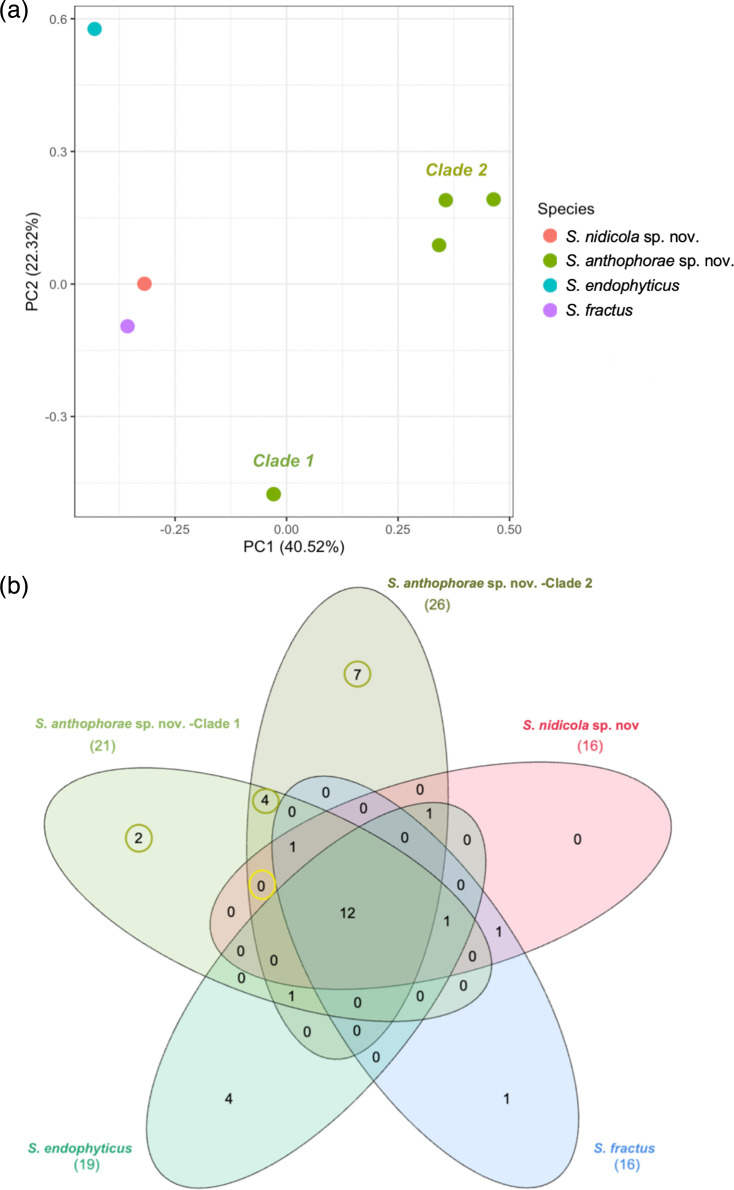
Overall composition of Biosynthetic Gene Clusters (BGCs) of *S. anthophorae* sp. nov., *S. nidicola* sp. nov. and closest relatives. (**a**) Principal Component Analysis (PCA) plot of BGC composition, showing distinction of BGC composition based both on species and on subclade. Analysis was limited to differentially present BGCs. Colour represents actual or proposed species, labels indicate clade within *S. anthophorae* sp. nov. BH034^T^ and BH097 are identical in BGC composition and thus the points are directly overlapping. PCA made with prcomp() in R ‘stats’ package (scale=T). (b) Venn diagram showing total differentiating and shared BGCs between bee isolates and close relatives (*S. anthophorae* sp. nov., two clades, *S. nidicola* sp. nov., *S. endophyticus* and *S. fractus*). Total number of BGCs in each species or group indicated in parentheses under name. *S. anthophorae* sp. nov. as a species (clades 1 and 2) has the most unique BGCs at 13* (circled in green) when compared to *S. endophyticus* YIM 65594^T^ and *S. fractus* MV32^T^. Clade 1 represents BH034T and BH097, clade 2 represents BH055, BH104 and BH105. Unique BGCs were not necessarily found in all strains within the clade. There are no BGCs that specifically distinguish all bee isolates from the other closest species (circled in yellow). *One of these 13 overlaps with a BGC found in *S. kunmingensis* DSM 41681^T^ (not shown), leaving 12 unique BGCs when compared to the three closest relatives (see Table S3). Made with InteractiVenn [68].

AntiSMASH was used to detect and identify BGCs in the genomes of these new isolates in comparison to those of close relatives (Fig. 3, Table S3) [49]. Given an annotated genome (Prokka), antiSMASH searches and annotates BGCs based on known arrangements and gene associations, as well as certain domain traits and comparison to characterized BGCs. This allows for the prediction of the type of metabolites the cluster may produce and provides a similarity score between each predicted cluster and its closest known cluster. The related *Streptomyces* genomes which were used for reference and comparison were *S. fractus* MV32^T^, *S. endophyticus* YIM 65594^T^, *S. kunmingensis* DSM 41681^T^ and one undescribed *Streptomyces* isolate from compost (NBC00311)*,* which was identified as potentially related via 16S SSU blast and ANI.

*S. anthophorae* sp. nov. strains (BH034^T^, BH055, BH097, BH104 and BH105) had between 21 and 24 BGCs in their genomes, while *S. nidicola* sp. nov. (BH106^T^) had 16 BGCs. *S. fractus* MV32^T^ also had 16 BGCs (15 of the 16 were the same as in BH106^T^); *S. endophyticus* YIM 65594^T^ had 19 BGCs (Fig. 3, Table S3). The BGC composition clusters by clade within *S. anthophorae* sp. nov. and *S. fractus* MV32^T^ closely resembles *S. nidicola* in composition (Fig. 3a); no unique BGCs were found in the genome of *S. nidicola* sp. nov. (BH106^T^). However, the *S. anthophorae* strains, taken together, contained 12 unique BGCs not found in their close relatives (*S. fractus* MV32^T^, *S. endophyticus* YIM 65594^T^ and *S. kunmingensis* DSM 41681^T^). Of these 12 unique BGCs, 10 have less than 50% similarity to any known cluster, and 8 have less than 25% similarity with any known cluster (Table S3). The unique BGCs span several BGC types – as characterized by antiSMASH, these were one butyrolactone, one lanthipeptide, three non-ribosomal peptide synthase (NRPS) or NRPS-like, one redox-cofactor, one regioselectivity recognizing element-containing, two type 2 polyketide synthase (T2 PKS), one type 3 polyketide synthase (T3 PKS), one terpene and one thioamitide class. Three of the 12 were found uniformly in all of the *S. anthophorae* sp. nov. strains, while the others, in most cases, belonged to one of the two subclades specifically. Seven BGCs were unique to clade 2 (BH055/BH104/BH105), but only three of these were found in all three of the strains; the others were uniquely present in only one or two of the three strains in the clade (Table S3). Two BGCs were unique to *S. anthophorae* clade 1 (BH034^T^/BH097), which were identical in BGC composition (Fig. 3b).

### Phenotypic differences

#### Morphology

##### *S. anthophorae* sp. nov.

**Fig. 4.**
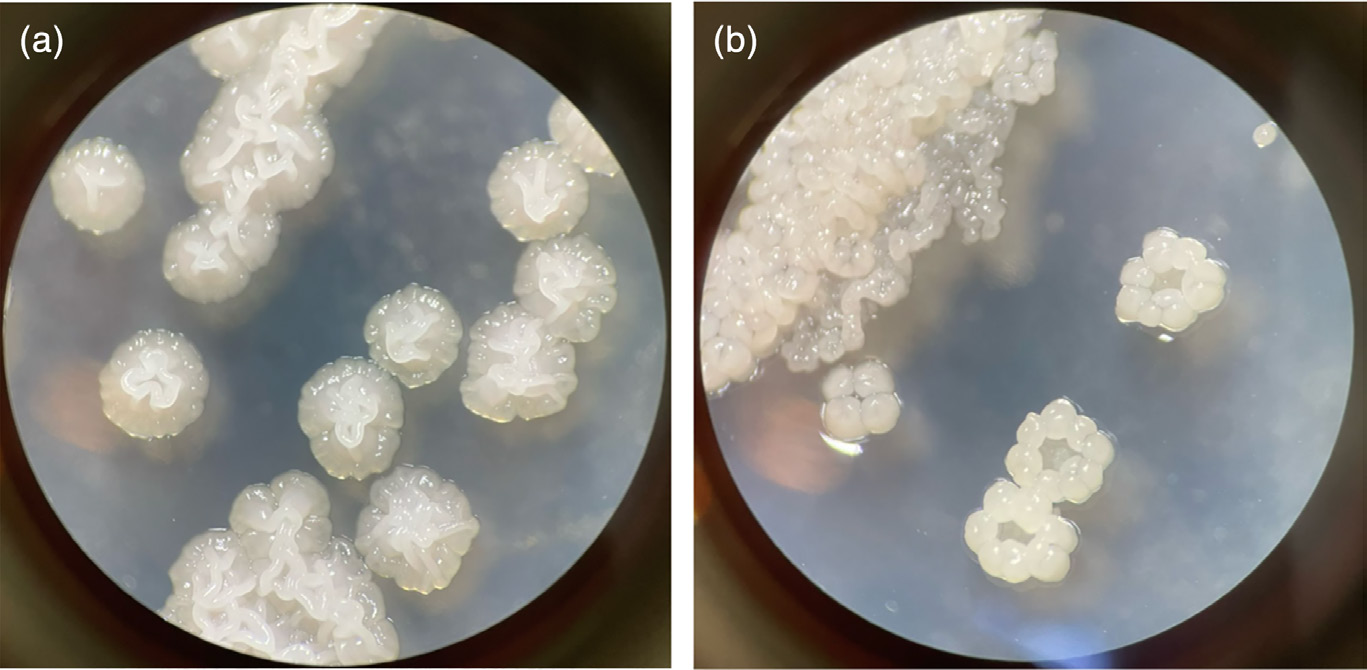
Colony appearance of newly described *Streptomyces* species. (**a**) *S. anthophorae* sp. nov. BH034^T^ and (b) *S. nidicola sp. nov*. BH106^T^ on TSA media after 4 days of growth (prior to pigment release) at 28 °C. Images taken at 30× on dissection microscope with a black background to enhance contrast.

On TSA solid media, colonies are irregular in shape and have lobate edges (Fig. 4a). The surface ranges between tightly wrinkled at the centre and flat with some wrinkles at the edge. Colonies are opaque, with a matte surface and viscoelastic texture, which tends to form a glob when picking colonies. Colonies are yellowish white to pale yellowish pink and secrete light to moderate yellowish brown diffusible pigments (Table 3). Odour is classic of geosmin production, earthy and intense. Sporulation was not observed on TSA but was observed on OA and MYM.

**Table 3.** Physiological characterization of bee-associated *Streptomyces* isolates representing new species and close relatives Rows represent each strain or species and selected differential characteristics; +, positive; +/−, weak; −, not observed; nd, no data. All data collected for this publication unless indicated with citation. See Methods for details.

Strain or species	Diffusible pigment (TSA)	Growth at (°C)						Growth with NaCl			Spore formation			pH					Frag. in liquid TSB	Geosmin production (TSA)
		5	25	28	37	40	42	5%	10%	15%	TSA	OA	MA	4.0	5.0	7.0	9.0	10.0		
BH034^T^ (*S. anthophorae* sp. nov.)	+/−*	+/−	+	+	+	+	+	+	+	−	−	+	−	−	+/−	+	+	+/−	+	+
BH055 (*S. anthophorae* sp. nov.)	+	nd	+	+	+	+	nd	+	+	−	−	+	+	nd	nd	nd	nd	nd	+	+
BH097 (*S. anthophorae* sp. nov.)	+	nd	+	+	+	+	nd	+	+	−	−	+	−	nd	nd	nd	nd	nd	+	+
BH104 (*S. anthophorae* sp. nov.)	+	nd	+	+	+	+	nd	+	+	−	−	+	−	nd	nd	nd	nd	nd	+	+
BH105 (*S. anthophorae* sp. nov.)	+	nd	+	+	+	+	nd	+	+	−	−	+/−	nd	nd	nd	nd	nd	nd	+	+
BH106^T^ (*S. nidicola* sp. nov.)	+	+/-	+	+	+	+	+	+	+	−	−	−	−	−	−	+	+/−	−	+	+/−
*S. fractus* MV32^T^	−	−	+	+	+	+	+	+	+	−	−	−	−	−	−	+	+/−	−	+	+
*S. endophyticus* YIM 65594^T^	− [16]	− [16]	+ [16]	+ [16]	+ [16]	− [16]	nd	+ [17]	+/− [17]	nd	nd	nd	nd	−	− [16]	+	+ [16]	nd	− [17]	nd
*S. kunmingensis* DSM 41681^T^	−	−	+	+	+	+	−	+/− [17]	+/− [17]	−	−	+	+	−	+	+	+/−	+/−	−	+
*S. variegatus* B-16380	−†	−	+	+	−	nd	nd	nd	−	nd	−	+	+	nd	nd	nd	nd	nd	−	+

* Visible red and green pigments on OA media, but they do not diffuse into the surrounding agar.

† Once made yellow exudate on colony surface in response to fungi.

On MYM solid media, colonies are entire with slightly ciliate edges. Dense colonies grow together to form irregular edges that curl back up and over the colonies, away from the media. The surface is yellowish white, somewhat matte, bumpy and complex with peeling back of edges. Within several days to a week, the surface becomes a matte white with sporulation. Colonies are firm and able to be scraped from the agar surface, with some breakage.

In liquid SGM, *S. anthophorae* initially grows in classical *Streptomyces* form, forming small fluffy spheres. However, after 2–3 days, the filaments fragment into smaller pieces and fall out of solution, forming a pellet.

##### *S. nidicola* sp. nov.

On TSA solid media, colonies are irregular in shape and have lobate edges that tend to be raised from the media surface on the edges, creating a ‘donut’-like appearance of early colonies, which then grow together (Fig. 4b). For older colonies or lawns, the surface ranges between tightly wrinkled (centre) and flat with raised wrinkled edges. Colonies are opaque, pale yellowish pink in colour, with matte surface and viscous to hard texture; they tend to break apart when picked. Colonies secrete pale pink to light yellowish brown diffusible pigments (Table 3). A distinct odour is often absent, but occasionally weakly earthy, suggesting geosmin production.

On MYM solid media, colonies are entire with slightly ciliate edges. Dense colonies grow together to form irregular edges which curl back up and over the colonies. Surface is pale yellowish pink, matte, bumpy and complex with peeling back of edges. Spore formation was not observed on any media tested (TSA, OA and MYM).

In liquid SGM, *S. nidicola* sp. nov. initially grows in classical *Streptomyces* form, forming small fluffy spheres. However, after 2–3 days, the filaments fragment into smaller pieces and fall out of solution.

### Physiology

Noticeable diffusible pigments were only produced by the bee-isolated *Streptomyces* strains, with *S. anthophorae* sp. nov. producing light to moderate yellowish brown pigmentation to various degrees and *S. nidicola* sp. nov. producing a pale pink to light yellowish brown pigment on TSA. *S. fractus* MV32^T^ and *S. kunmingensis* DSM 41681^T^ did not produce noticeable diffusible pigmentation on TSA. Salt tolerance also differentiated the species, with all *S. anthophorae* sp. nov. strains being salt tolerant (10%) along with *S. fractus* MV32^T^*,* whereas *S. endophyticus* YIM 65594^T^, *S. kunmingensis* DSM 41681^T^ and *Streptomyces variegatus* B-16380 are not salt tolerant, or grew only weakly (Table 3) [16]. None of the tested strains tolerated 15% NaCl. Only the new isolates as well as *S. fractus* MV32^T^ fragmented after several days in liquid TSB media. Both new type strains (BH034^T^ and BH106^T^) grew at 42 °C, as did *S. fractus* MV32^T^, but not *S. kunmingensis* DSM 41681^T^; *S. endophyticus* YIM65594^T^ and *S. variegatus* B-16380 fail to grow at 37 °C [17]. Both new type strains (BH034^T^ and BH106^T^) also grew weakly at 5 °C, *S. fractus* MV32^T^ and *S. kunmingensis* DSM 41681^T^ did not, and *S. endophyticus* YIM 65594^T^ is unable to grow at 4 °C per [16]. Spore formation was observed for all *S. anthophorae* sp. nov. strains on OA but was not observed for *S. nidicola* sp. nov. nor for *S. fractus* MV32^T^ on any tested media (Table 3). Growth in TSB with pH adjustment to pH 4.0, 5.0, 7.0, 9.0 and 10.0 showed that *S. anthophorae* sp. nov. BH034^T^ grew weakly at pH 5.0 and 10.0 and normally between 7.0 and 9.0. *S. fractus* MV32^T^ and *S. nidicola* sp. nov. BH106^T^ grew normally at pH 7.0 and weakly at pH 9.0 and did not grow at the other tested pH levels.

## Substrate utilization and resistance

Type strains of each proposed new species (BH034^T^ and BH106^T^) as well as closely related *S. fractus* MV32^T^ and *S. kunmingensis* DSM 41681^T^ were tested for growth on 71 different substrates using Biolog GenIII microplates. These were compared to the literature for *S. endophyticus* YIM 65594^T^ [16]. Key differences between the two bee-isolated type strains and the closely related *S. fractus* MV32^T^ and *S. kunmingensis* DSM 41681^T^ were that neither bee isolate was able to grow on pectin, whereas both *S. fractus* MV32^T^ and *S. kunmingensis* DSM 41681^T^ were able to grow weakly on it. The two bee-isolated type strains were able to grow only weakly or not at all on methyl pyruvate, l-pyroglutamic acid and l-fucose, whereas both *S. fractus* MV32^T^ and *S. kunmingensis* DSM 41681^T^ were able to grow normally on these substrates. BH034^T^ was differentiated from *S. endophyticus* YIM 65594^T^ literature by the ability to utilize sucrose, and BH106^T^ was differentiated by the inability to utilize d-maltose [16].

There were also differences between the type strains of each proposed new species (BH034^T^ and BH106^T^). Sucrose, d-maltose, d-fucose, d-fructose 6-PO4, l-histidine and stachyose were utilized by BH034^T^ but not BH106^T^. BH106^T^ was able to utilize *γ*-amino-butyric acid (GABA), l-alanine, mucic acid, acetic acid and formic acid, whereas BH034^T^ was only able to weakly grow on these substrates. See Table 4 for select distinguishing characteristics and Table S4 for full profiles.

**Table 4.** Substrate utilization differs among bee-associated *Streptomyces* and close relatives Select substrates showing differential utilization. Growth was assessed using single strains inoculated into Biolog GenIII microplate with 1% NaCl solution. Growth indicated by (+), weak growth (w) and no growth (−).

Substrate	*S. anthophorae* sp. nov. BH034^T^	*S. nidicola* sp. nov. BH106^T^	*S. fractus* MV32^T^	*S. kunmingensis* DSM 41681^T^	*S. endophyticus* YIM 65594^T^ [16]
Pectin	−	−	w	w	nd
l-Pyroglutamic acid	w	w	+	+	nd
Methyl pyruvate	w	w	+	+	nd
l-Fucose	w	−	+	+	nd
d-Maltose	+	−	+	+	+
Sucrose	+	−	+	+	−
d-Fucose	+	−	+	w	nd
d-Fructose 6-PO4	+	−	+	w	nd
l-Histidine	+	−	w	+	nd
Stachyose	+	−	+	w	nd
*γ*-Amino-butyric acid (GABA)	w	+	+	w	nd
l-Alanine	w	+	w	+	+
Mucic acid	w	+	+	w	nd
Acetic acid	w	+	w	+	nd
Formic acid	w	+	+	−	nd

## Chemotaxonomy

The major cellular fatty acids (>10%) in strain BH034^T^ were iso-C_16 : 0_ (25.1%), anteiso-C_15 : 0_ (19.9%) and iso-C_15 : 0_ (16.2%), whereas in BH106^T^, the major cellular fatty acids were anteiso-C_15 : 0_ (25.4%), anteiso-C_17: 0_ (20.1%), iso-C_17 : 0_ (13.7%) and iso-C_15 : 0_ (12.9%) (Table 5). The fatty acid patterns of both strains correspond to fatty acid pattern 2 c of Kroppenstedt (1985), indicating *Streptomyces* genus, but differ from the reported profiles of the type strains of the closest relatives *S. fractus* MV32^T^ and *S. endophyticus* YIM 65594^T^. All four strains share anteiso-C_15 : 0_ as a major fatty acid. The other major fatty acids distinguish one or more strains from the others. BH034^T^ and BH106^T^ are distinguishable from each other and the closely related species by fatty acid composition. The cell walls of BH034^T^ and BH106^T^ contained ll-diaminopimelic acid.

**Table 5.** Cellular fatty acid profiles and cell-wall ll-DAP of the bee-isolated type strains BH034ᵀ and BH106ᵀ compared with their closest related species, *S. fractus* MV32^T^ [17] and *S. endophyticus* YIM 65594^T^ [16] All fatty acids for which any individual strain had over 2% are shown. Major fatty acids, defined as those comprising >10% of total fatty acids, are indicated by bold text. ll-DAP is present in all strains.

Fatty acid/component	*S. anthophorae* sp. nov. BH034^T^	*S. nidicola* sp. nov. BH106^T^	*S. fractus* MV32^T^	*S. endophyticus* YIM 65594 ^T^
15:0 anteiso	**19.9**	**25.4**	**36.5**	**34.2**
17:0 anteiso	9.7	**20.1**	**20.3**	**28.3**
16:0 iso	**25.1**	7.3	**15.6**	**20.9**
15:0 iso	**16.2**	**12.9**	6.3	2.9
17:0 iso	7.4	**13.7**	3.1	2.3
14:0 iso	8.2	1.9	1.8	1.6
16:0	4.1	3.8	3.0	−
18:1 iso *ω*6c	1.5	3.6	−	−
17:1 anteiso *ω*9c	−	−	4.1	2.9
18:3 *ω*6c (6,9,12)	1.5	2.3	−	−
16:1 iso H	0.4	0.1	2.8	1.4
ll-DAP	*+*	*+*	*+*	*+*
*Source*	*This paper*	*This paper*	[17]	[16]

## Discussion

*Streptomyces* spp. are commonly associated with plants and animals [8,10,56, 57]. These relationships are often mutualisms, with the host providing a consistent growth environment and transmission of the bacteria between generations and the *Streptomyces* providing protection from infection through production of secondary metabolites [10,11, 58, 59]. In previous work, we found that *Streptomyces* is present throughout the development of the bee *A. bomboides* but is especially abundant during overwintering, when the bee faces heightened pathogen pressure [14]. Four of the six strains described here have been shown to inhibit the growth of bee fungal pathogens [14], so they may be involved in the defence of the developing bees against microbial pathogens.

Genomic analysis showed that the bee isolates described here separate into two novel species, one being a clade of five closely related isolates, here named *S. anthophorae* sp. nov., and the other consisting of one isolate, here named *S. nidicola* sp. nov. This finding was supported by whole-genome comparison methods (ANI, dDDH), as well as MLSA and whole-genome trees, which confirmed the strong support for separation of these isolates from known species and into these two new species. The two species most closely related to the new isolates are *S. endophyticus* YIM 65594^T^ and *S. fractus* MV32^T^ (Fig. 1). Both are associated with a host, with *S. endophyticus* being associated with the roots of *Artemisia annua* and *S. fractus* being associated with the gut of the black mound termite (*A. hastatus*) [16,17]. The genome of *S. endophyticus* YIM 65594^T^ was available on the NCBI, and the physiology was described [16]; thus, we used only the genome and literature review for comparisons. The genome of *S. fractus* was not available, but as the type strain (MV32^T^) was freely available from the USDA ARS Culture Collection, we ordered, sequenced and characterized it alongside the *S. anthophorae* and *S. nidicola* strains. This allowed for full genomic comparisons of these two most closely related species with the bee-isolated strains. Interestingly, the tolerance of salt and high temperature exhibited by the bee-isolated strains was most like that of *S. fractus* MV32^T^, which was also isolated from a soil-dwelling insect (black mound termite). The similarities also included fragmentation in liquid media [17].

*Streptomyces* are known for their production of secondary metabolites, and analysis of BGC composition in the isolate genomes as compared to the most closely related species showed that *S. anthophorae* sp. nov. strains have unique BGCs as compared to close relatives. The BGCs are distinct among the two clades within *S. anthophorae*, with clade 2 (BH055/BH104/BH105) having the highest number of unique BGCs, though they were not all consistently found in all three strains (Fig. 3). In previous work, we found that BH034^T^ and BH097 (clade 1) showed higher inhibition of the fungal bee pathogen *Ascosphaera* apis than BH055 and BH104 (clade 2) [14], which indicates that one or both BGCs that differentiate the clades may encode the biosynthetic machinery for an antifungal compound.

### Significance

Pathogenic fungi threaten human health, as well as the productivity of animals and plants cultivated for agriculture [60]. Infection and mortality from pathogenic fungi are increasing threat due to emerging fungal species, increasing resistance to existing antifungal compounds and limited antifungal modes of action available for therapeutic use [61]. Much effort has been dedicated to antifungal natural products discovery from soil-dwelling bacteria, marine organisms and other potential sources of novel chemistries [8,62,64]. However, isolation campaign success has been limited by context-specific metabolite production, compound rediscovery and off-target toxicity for eukaryotic host animals [65]. To overcome these challenges, host-microbe symbiotic systems have been proposed as platforms for antifungal chemistry discovery, but few systems have been leveraged to date; insect-microbe systems specifically have been shown to hold exceptional potential for novel, safe and effective antifungal chemistry [8,10, 63]. These genomes and analysis will allow further exploration of previously undescribed metabolite diversity and may yield novel antifungal agents that could be leveraged for therapeutic use.

## Description of *Streptomyces anthophorae* sp. nov.

*Streptomyces anthophorae* sp. nov. (an.tho’pho.rae. N.L. gen. n. *anthophorae*, of the solitary bee *Anthophora bomboides*).

The type strain is BH034^T^ and was isolated from a pollen provision in a brood cell of developing *A. bomboides. S. anthophorae* sp. nov. is a Gram-positive, filamentous bacterium [66]. It exhibits fragmenting growth in liquid media and inhibits the growth of several bee-associated filamentous fungi [14]. The temperature range for growth is 25–42 °C, with weak growth occurring at 5 °C and optimal growth between 28 and 37 °C on TSA or in liquid SGM. The pH range for growth is 5.0–10.0, optimum pH 7.0. It occasionally produces light to moderate yellowish-brown diffusible pigment on solid media and is halotolerant to 10% NaCl. It consistently produces white spores on OA and is catalase positive.

Able to utilize d-raffinose, alpha-d-glucose, gelatin, Tween 40, dextrin, alpha-d-lactose, d-mannose, d-mannitol, glycyl-l-proline, d-galacturonic acid, d-maltose, d-melibiose, d-fructose, d-arabitol, l-galactonic acid lactone, alpha-hydroxybutyric acid, d-trehalose, beta-methyl-d-glucoside, d-galactose, l-arginine, d-gluconic acid, l-lactic acid, beta-hydroxy-d,l- butyric acid, d-cellobiose, d-salicin, glycerol, l-aspartic acid, d-glucuronic acid, citric acid, alpha-keto butyric acid, gentiobiose, *N*-acetyl-d-glucosamine, d-fucose, d-glucose 6-PO4, l-glutamic acid, sucrose, d-fructose 6-PO4, l-histidine, d-malic acid, propionic acid, d-turanose, *N*-acetyl-d-galactosamine, l-rhamnose, quinic acid, l-malic acid, stachyose, N-acetyl-neuraminic acid, inosine, l-serine, d-saccharic acid and bromo-succinic acid. Able to weakly utilize d-sorbitol, methyl pyruvate, y-amino-butyric acid, l-alanine, d-lactic methyl ester, myo-inositol, glucuronamide, alpha-keto-glutaric acid, acetoacetic acid, l-fucose, mucic acid, d-aspartic acid, l-pyroglutamic acid, acetic acid, d-serine and formic acid. Unable to utilize pectin, p-hydroxyphenylacetic acid, 3-methyl glucose or *N*-acetyl-beta-d-mannosamine. The major cellular fatty acids (>10%) are iso-C_16 : 0_, anteiso-C_15 : 0_ and iso-C_15 : 0_. Whole-cell hydrolysates contain ll-diaminopimelic acid. The genome of the type strain (BH034^T^) is 9.62 Mb with a G+C content of 71 mol% and 8,716 predicted genes. The type strain (BH034^T^) is available under collection numbers NRRL B-65741 and DSM 119658 from the USDA ARS Collection and the DSMZ German Collection of Microorganisms and Cell Cultures, respectively. The genome is available on the NCBI (JBLWQQ000000000).

## Description of *Streptomyces nidicola* sp. nov.

*Streptomyces nidicola* sp. nov. (ni.di’co.la. L. masc. n. *nidus*, nest; L. masc./fem. suff. -*cola* (from L. masc./fem. n. *incola*), inhabitant, dweller; N.L. masc. n. *nidicola*, nest dweller, referring to the isolation of the type strain from a solitary bee nest)

The type strain is BH106^T^ and was isolated from an *A. bomboides* pupa within a brood cell. *S. nidicola* sp. nov. is a Gram-positive, filamentous bacterium that exhibits fragmenting growth in liquid media [66]. The temperature range for growth is 25–42 °C, with weak growth occurring at 5 °C and optimal growth between 28 and 37 °C on TSA or in liquid SGM. The pH range for growth is 7.0–9.0, optimum pH 7.0. Species produces pale pink to light yellowish brown diffusible pigment on solid TSA media and can weakly grow in 10% NaCl. It is catalase positive and did not produce spores on any tested media type. Able to utilize alpha-d-glucose, gelatin, Tween 40, dextrin, alpha-d-lactose, d-mannose, d-mannitol, glycyl-l-proline, y-amino-butyric acid, d-fructose, d-arabitol, l-alanine, l-galactonic acid lactone, d-trehalose, d-galactose, l-arginine, d-gluconic acid, beta-hydroxy-d,l- butyric acid, d-cellobiose, d-salicin, glycerol, l-aspartic acid, d-glucuronic acid, citric acid, alpha-keto butyric acid, gentiobiose, *N*-acetyl-d-glucosamine, l-glutamic acid, mucic acid, d-malic acid, propionic acid, *N*-acetyl-d-galactosamine, l-rhamnose, quinic acid, l-malic acid, acetic acid, *N*-acetyl-neuraminic acid, inosine, l-serine, d-saccharic acid and formic acid. Able to weakly utilize d-raffinose, d-sorbitol, d-galacturonic acid, methyl pyruvate, d-melibiose, alpha-hydroxybutyric acid, beta-methyl-d-glucoside, l-lactic acid, d-glucose 6-PO4, glucuronamide, alpha-keto-glutaric acid, acetoacetic acid, d-turanose, d-aspartic acid, l-pyroglutamic acid and bromo-succinic acid. Unable to utilize pectin, p-hydroxyphenylacetic acid, d-maltose, d-lactic methyl ester, myo-inositol, 3-methyl glucose, d-fucose, sucrose, *N*-acetyl-beta-d-mannosamine, l-fucose, d-fructose 6-PO4, l-histidine, stachyose or d-serine. The major fatty acids are anteiso-C_15 :0_, anteiso-C_17:0_, iso-C_17 :0_ and iso-C_15 :0_. Whole-cell hydrolysates contain ll-diaminopimelic acid.

The genome of the *S. nidicola* sp. nov. type strain (BH106^T^) is 9.4 Mb with a G+C content of 70.7% and 8,426 predicted genes. The type strain (BH106^T^) is available under collection numbers NRRL B-65742 and DSM 119659 from the USDA ARS Collection and the DSMZ German Collection of Microorganisms and Cell Cultures, respectively. The genome is available on the NCBI (JBNBPI000000000).

## Supplementary material

10.1099/ijsem.0.007029 Uncited Supplementary Material 1.

10.1099/ijsem.0.007029 Uncited Supplementary Material 2.

## Abbreviations

ANI: average nucleotide identity
ASV: amplicon sequencing variant
BGC: biosynthetic gene cluster
dDDH: digital DNA–DNA hybridization
GBDP: Genome blast Distance Phylogeny
MAFFT: Multiple Alignment using Fast Fourier Transform
MLSA: multilocus sequence alignment
MYM: maltose yeast media
NJ: neighbour-joining
NRPS: non-ribosomal peptide synthase
OA: oatmeal agar
SGM: supplemented Grace’s media
TSA: tryptic soy agar
