# Supplementary material for: Streptomyces anthophorae sp. nov. and Streptomyces nidicola sp. nov., novel actinobacteria isolated from a solitary bee
Source: Int J Syst Evol Microbiol. 2026 Jan 29;76(1):007029. doi: 10.1099/ijsem.0.007029 (PMC12856498; doi:10.1099/ijsem.0.007029)
Supplement: Uncited Supplementary Material 1. [file ijsem-76-07029-s001.pdf]

Supplementary Information

Title: *Streptomyces anthophorae* sp. nov. and *Streptomyces nidicola* sp. nov., novel actinobacteria isolated from a solitary bee

Authors: Shawn M Christensen (1), Martin Kaltenpoth (2), Heiko Vogel (2), Rachel L Vannette (1)

Affiliations: 1) Department of Entomology and Nematology, University of California- Davis, Davis, CA, United States, 2) Department of Insect Symbiosis, Max Planck Institute for Chemical Ecology, Jena, Germany

Table S1: Media recipes

Tryptic Soy Agar (TSA):

- 1000 mL DI H2O
- 15 g tryptone
- 15 g agar
- 5 g soytone
- 5 g NaCl
- 50g fructose
- 1mL cycloheximide (stock 100 mg/mL in methanol, added to media after autoclaving)

Maltose Yeast Medium (MYM):

- 1000 mL DI H2O
- 4 g maltose
- 4 g yeast extract
- 10 g malt extract
- 15 g agar

Oatmeal Agar (OA):

- 1000 mL DI H2O
- 40 g pulverized oatmeal
- 15 g agar

Yeast Media (YM):

- 1000 mL DI H20
- 3 g malt extract
- 5 g peptone
- 10 g glucose
- 10 g agar
- 3 g yeast extract
- 500 µL chloramphenicol (stock 100 mg/mL in methanol, added to media after autoclaving)

Table S2 -Digital DNA-DNA hybridization

dDDH table from DSMZ server showing dDDH values between new isolates (BH034-BH106) and known species. d0 value followed by the confidence interval in brackets.

	BH097	BH055	BH104	BH105	BH106 T	*S. fractus* MV32 T	*S. endophyticus* YIM 65594 T	*S.kunmingensis* DSM 41681 T
BH034 T	99.8 [99.6 99.9]	78.8 [74.9 82.3]	69.6 [65.7 73.3]	73.5 [69.5 77.1]	64.5 [60.7 68.2]	55.9 [52.3 59.4]	65.9 [62.1 69.6]	32.5 [29.1 36.1]
BH097	x	78.4 [74.4 81.9]	69.2 [65.3 72.8]	73.1 [69.1 - 76.7]	64.2 [60.4 67.8]	55.6 [52.1 59.1]	65.6 [61.8 69.3]	32.4 [29.1 36.0]
BH055		x	86.4 [82.8 89.4]	90.9 [87.7 93.3]	65.9 [62.1 69.5]	54.8 [51.3 - 58.3]	64 [60.2 67.6]	32.6 [29.3 36.2
BH104			x	80.3 [76.4 83.7]	60 [56.3 63.5]	49.8 [46.4 53.3]	58 [54.4 61.5]	31.8 [28.5 35.4]
BH105				x	64.9 [61.1 68.6]	55.6 [52.0 59.1]	65.4 [61.6 69.1]	32.5 [29.1 36.1]
BH106 T					x	60.3 [56.6 63.8]	67.7 [63.9 71.4]	32.9 [29.5 36.5]

Table S3- Biosynthetic gene clusters

All biosynthetic gene clusters in the analyzed genomes listed down left side, showing antiSMASH assigned type and most similar BGC cluster ('sim_cluster'). The following columns represent each genome (bold), with colored/filled areas indicating that the BCG in that row is present in that genome, the percentage is the assigned similarity to the closest known cluster. After the genome columns, the next three columns (13-15) indicate whether a BGC (row) is unique to *S. anthophorae*, and then to which clade (if it is unique to only one of the clades). These unique BCGs (their closest known cluster) is shown again in the last column. The related Streptomyces genomes which were used for reference and comparison were: *S. fractus* MV32 ^{T}*, S. endophyticus* YIM 65594 ^{T}*, S. kunmingensis* DSM 41681^{T}, and one undescribed *Streptomyces* isolate from compost (NBC00311), which was identified as potentially related via 16S SSU BLAST and ANI.

*(continued) 5-acetyl-5,10-dihydrophenazine-1-carboxylic acid/5-(2-hydroxyacetyl)-5,10-dihydrophenazine-1-carboxylic acid/endophenazine A1/endophenazine F

Type	*Sim_cluster*	**BH034**	**BH097**	**BH055**	**BH104**	**BH105**	**BH106**	**NBC00311**	***S. endophyticus***	***S. fractus***	***S. kunmingensis* 41681**	unique to *S. anthophorae*?	unique to clade 1?	unique to clade 2?		Less than 50% similarity to known clusters?	Less than 25% similarity to known clusters?
betalactone	betalactone										0						
butyrolactone	lactonamycin			10%	10%	8%					8%						
	amipurimycin	6%	6%	6%	6%	6%									amipurimycin	y	y
ectoine	ectoine	100%	100%	100%	100%	100%	100%	100%	100%	100%	100%						
hydrogen-cyanide	aborycin	14%	14%	14%	14%	14%	14%	14%	14%	14%							
lanthipeptide-class-i	lanthipeptide-class-i	0	0	0		0		0	0		0						
lanthipeptide-class-iii	lanthipeptide-class-iii								0								
	informatipeptin	42%	42%	42%	42%	42%									informatipeptin	y	n
	AmfS										80%						
lassopeptide	SRO15-2005										87%						
	lassopeptide										0						
melanin	istamycin	4%	4%	4%	4%	4%		4%			5%						
NAPAA	ε-Poly-L-lysine	100%	100%	100%	100%	100%	100%	100%	100%	100%	100%						
	stenothricin							13%	13%		13%						
NI-siderophore	kinamycin	22%	22%	22%	22%	22%	22%	22%	22%	22%	22%						
	desferrioxamin B/desferrioxamine E	100%	100%	100%	100%	100%	100%	100%	100%	100%	100%						
NRP-metallophore,NRPS	paenibactin				83%	83%	83%	83%	83%		83%						
NRPS	WS9326	7%	7%												WS9326	y	y
	vazabitide A			15%	15%	15%									vazabitide A	y	y
	s56-p1								3%								
	omnipeptin										9%						
	NRPS										0						
NRPS-like	enduracididine										16%						
	bombyxamycin A/bombyxamycin B										11%						
NRPS-like,NRPS	thiocoraline			47%											thiocoraline	y	n
NRPS,lanthipeptide-class-ii	omnipeptin										9%						
nucleoside	toyocamycin										30%						
other	tambjamine BE-18591							25%									
other,butyrolactone	5-acetyl-5,10-dihydrophenazine-1-car										26%						
PKS-like	colabomycin E						9%			6%							
PKS-like,butyrolactone	marineosin A/marineosin B							9%									
redox-cofactor	redox-cofactor	0	0				0	0	0	0							
	meridamycin			5%	5%	5%									meridamycin	y	y
	enduracididine									6%							
	calicheamicin										6%						
RiPP-like	triacsin C	6%	6%	6%	6%	6%	6%	6%	6%	6%							
	RiPP-like	0	0	0	0	0	0	0	0	0	0						
	hexacosalactone A	4%	4%	4%	4%	4%	4%	4%	4%	4%	4%						
RRE-containing	RRE-containing										0						
	frankiamicin	21%	21%	21%	21%	21%									frankiamicin	y	y
T1PKS	teicoplanin										3%						
	T1PKS										0						
	quinolidomicin A										22%						
	mediomycin A										36%						
	filipin										38%						
	desertomycin B/desertomycin A/des										12%						
T1PKS,oligosaccharide	ibomycin										31%						
T2PKS	WS-5995 D/WS-5995 B/WS-5995 C/S	21%	21%												WS-5995 D/WS	y	y
	rubiginone A2/rubiginone J/rubigino										41%						
	nenestatin			50%	50%	50%									nenestatin	n	n
	isofuranonaphthoquinone							95%									
T2PKS,butyrolactone	allocycline							56%									
T2PKS,terpene	lugdunomycin								62%								
T3PKS	violapyrone B	28%	28%	28%		28%	28%	28%	28%	28%							
	T3PKS										0						
T3PKS,NRP-metallophore,NI	paenibactin			83%											paenibactin	n	n
T3PKS,phenazine	endophenazine A/endophenazine B										77%						
terpene	terpene										0						
	hopene	84%	84%	84%	84%	84%	84%	84%	84%	84%	84%						
	geosmin	100%	100%	100%	100%	100%	100%	100%	100%	100%	100%						
	ebelactone			8%		8%									ebelactone	y	y
	carotenoid										63%						
	albaflavenone	100%	100%	100%	100%	100%	100%	100%	100%	100%	100%						
	2-methylisoborneol	100%	100%		100%		100%	100%		100%	100%						
thioamitides	thioamitides				0										thioamitides	y	y
**TOTAL BGCs**		**21**	**21**	**24**	**22**	**23**	**16**	**22**	**19**	**16**	**40**	**12**	**2**	**7**			

Table S4 – Substrate utilization

Also provided as additional excel file. Growth on 71 substrates using Biolog GenIII microplate in 1% NaCl. Growth (+), weak growth (w), no growth (-).

Broad Class	Compound Class	Compound	*S. anthophorae sp. nov BH034 T*	*S. nidicola sp. nov BH106 T*	*S. fractus MV32 T*	*S. kunmingensis NBRC 14463 T*
Amino Acid/Peptide	Amino acid	D-Aspartic acid	w	w	w	w
Amino Acid/Peptide	Amino acid	D-Serine	w	-	w	w
Amino Acid/Peptide	Amino acid	L-Alanine	w	+	w	+
Amino Acid/Peptide	Amino acid	L-Arginine	+	+	+	w
Amino Acid/Peptide	Amino acid	L-Aspartic acid	+	+	+	w
Amino Acid/Peptide	Amino acid	L-Glutamic acid	+	+	+	+
Amino Acid/Peptide	Amino acid	L-Histidine	+	-	w	+
Amino Acid/Peptide	Amino acid	L-Serine	+	+	+	+
Amino Acid/Peptide	Amino acid derivative	L-Pyroglutamic acid	w	w	+	+
Amino Acid/Peptide	Amino acid derivative	γ-Amino-butyric acid (GABA)	w	+	+	w
Amino Acid/Peptide	Dipeptide	Glycyl-L-proline	+	+	+	+
Carbohydrate	Amino sugar	N-Acetyl-D-galactosamine	+	+	+	+
Carbohydrate	Amino sugar	N-Acetyl-D-glucosamine	+	+	+	+
Carbohydrate	Amino sugar	N-Acetyl-β-D-mannosamine	-	-	w	-
Carbohydrate	Amino sugar derivative	N-Acetyl neuraminic acid (sialic acid)	+	+	+	-
Carbohydrate	Disaccharide	D-Cellobiose	+	+	+	+
Carbohydrate	Disaccharide	D-Maltose	+	-	+	+
Carbohydrate	Disaccharide	D-Melibiose	+	w	w	+
Carbohydrate	Disaccharide	D-Trehalose	+	+	+	+
Carbohydrate	Disaccharide	D-Turanose	+	w	w	-
Carbohydrate	Disaccharide	Gentiobiose	+	+	+	+
Carbohydrate	Disaccharide	Sucrose	+	-	+	-
Carbohydrate	Disaccharide	α-D-Lactose	+	+	+	+
Carbohydrate	Glycoside	D-Salicin	+	+	+	+
Carbohydrate	Glycoside	β-Methyl-D-glucoside	+	w	+	w
Carbohydrate	Modified monosaccharide	3-Methyl glucose	-	-	-	-
Carbohydrate	Monosaccharide	D-Fructose	+	+	+	+
Carbohydrate	Monosaccharide	D-Galactose	+	+	w	+
Carbohydrate	Monosaccharide	D-Mannose	+	+	+	+
Carbohydrate	Monosaccharide	α-D-Glucose	+	+	+	+
Carbohydrate	Monosaccharide (deoxy sugar)	D-Fucose	+	-	+	w
Carbohydrate	Monosaccharide (deoxy sugar)	L-Fucose	w	-	+	+

Carbohydrate	Monosaccharide (deoxy sugar)	L-Rhamnose	+	+	+	+
Carbohydrate	Oligosaccharide	D-Raffinose	+	w	+	+
Carbohydrate	Oligosaccharide	Stachyose	+	-	+	w
Carbohydrate	Phosphorylated sugar	D-Fructose 6-phosphate	+	-	+	w
Carbohydrate	Phosphorylated sugar	D-Glucose 6-phosphate	+	w	+	w
Carbohydrate	Sugar alcohol (polyol)	D-Arabitol	+	+	+	+
Carbohydrate	Sugar alcohol (polyol)	D-Mannitol	+	+	+	+
Carbohydrate	Sugar alcohol (polyol)	D-Sorbitol	w	w	w	-
Carbohydrate	Sugar alcohol (polyol)	Glycerol	+	+	+	+
Carbohydrate	Sugar alcohol (polyol)	myo-Inositol	w	-	w	-
Nucleoside/Nucleotide	Nucleoside	Inosine	+	+	+	+
Organic Acid	Amide derivative	Glucuronamide	w	w	w	w
Organic Acid	Aromatic organic acid	p-Hydroxyphenylacetic acid	-	-	-	-
Organic Acid	Dicarboxylic acid derivative	Bromo-succinic acid	+	w	+	w
Organic Acid	Hydroxy acid	L-Lactic acid	+	w	+	-
Organic Acid	Hydroxy acid	α-Hydroxybutyric acid	+	w	w	+
Organic Acid	Hydroxy acid	β-Hydroxy-D,L-butyric acid	+	+	+	+
Organic Acid	Keto acid	Acetoacetic acid	w	w	w	w
Organic Acid	Keto acid	α-Ketobutyric acid	+	+	+	+
Organic Acid	Keto acid (TCA cycle)	α-Ketoglutaric acid	w	w	w	w
Organic Acid	Keto acid derivative	Methyl pyruvate	w	w	+	+
Organic Acid	Organic acid (1C)	Formic acid	w	+	+	-
Organic Acid	Organic acid (cyclitol derivative)	Quinic acid	+	+	+	+
Organic Acid	Organic acid (sugar acid)	D-Gluconic acid	+	+	+	+
Organic Acid	Organic acid (TCA cycle)	Citric acid	+	+	+	+
Organic Acid	Organic acid (TCA cycle)	D-Malic acid	+	+	+	-
Organic Acid	Organic acid (TCA cycle)	L-Malic acid	+	+	+	+
Organic Acid	Organic acid (uronic acid)	D-Galacturonic acid	+	w	+	+
Organic Acid	Organic acid (uronic acid)	D-Glucuronic acid	+	+	+	+
Organic Acid	Organic acid ester	D-Lactic acid methyl ester	w	-	w	w
Organic Acid	Short-chain fatty acid	Acetic acid	w	+	w	+
Organic Acid	Short-chain fatty acid	Propionic acid	+	+	+	+
Organic Acid	Sugar acid (dicarboxylic acid)	D-Saccharic acid	+	+	+	w
Organic Acid	Sugar acid (dicarboxylic acid)	Mucic acid (galactaric acid)	w	+	+	w

Organic Acid	Sugar acid (lactone)	L-Galactonic acid lactone	+	+	+	+
Polymer/Complex	Complex polymer (detergent)	Tween 40	+	+	+	+
Polymer/Complex	Polysaccharide	Pectin	-	-	w	w
Polymer/Complex	Polysaccharide (oligoglucan)	Dextrin	+	+	+	+
Polymer/Complex	Protein / polymer	Gelatin	+	+	+	w
		negative control	-	-	-	-
		positive control	+	+	+	+
